# Effectiveness and comparative effectiveness of evidence-based psychotherapies for posttraumatic stress disorder in clinical practice

**DOI:** 10.1017/S0033291721001628

**Published:** 2023-01

**Authors:** Shira Maguen, Erin Madden, Nicholas Holder, Yongmei Li, Karen H. Seal, Thomas C. Neylan, Callan Lujan, Olga V. Patterson, Scott L. DuVall, Brian Shiner

**Affiliations:** 1 Mental Health Service, San Francisco VA Health Care System, San Francisco, CA; 2 Department of Psychiatry, University of California, San Francisco, San Francisco, CA; 3 Integrative Health Service, San Francisco VA Health Care System, San Francisco, CA; 4 Department of Medicine and Psychiatry, University of California, San Francisco, San Francisco, CA; 5 Department of Neurology, University of California, San Francisco, San Francisco, CA; 6 VA Informatics and Computing Infrastructure, VA Salt Lake City Health Care System, Salt Lake City, Utah; 7 Department of Internal Medicine, University of Utah, School of Medicine, Salt Lake City, Utah; 8 Mental Health Service, White River Junction VA Medical Center, and National Center for Posttraumatic Stress Disorder, Executive Division, White River Junction, VT; 9 Department of Psychiatry, Geisel School of Medicine at Dartmouth, Hanover, NH

**Keywords:** Veteran, Posttraumatic Stress Disorder, Effectiveness, Comparative Effectiveness, Evidence-based psychotherapy, cognitive processing therapy, prolonged exposure therapy

## Abstract

**Background:**

While evidence-based psychotherapy (EBP) for posttraumatic stress disorder (PTSD) is a first-line treatment, its real-world effectiveness is unknown. We compared cognitive processing therapy (CPT) and prolonged exposure (PE) each to an individual psychotherapy comparator group, and CPT to PE in a large national healthcare system.

**Methods:**

We utilized effectiveness and comparative effectiveness emulated trials using retrospective cohort data from electronic medical records. Participants were veterans with PTSD initiating mental healthcare (*N* = 265 566). The primary outcome was PTSD symptoms measured by the PTSD Checklist (PCL) at baseline and 24-week follow-up. Emulated trials were comprised of ‘person-trials,’ representing 112 discrete 24-week periods of care (10/07–6/17) for each patient. Treatment group comparisons were made with generalized linear models, utilizing propensity score matching and inverse probability weights to account for confounding, selection, and non-adherence bias.

**Results:**

There were 636 CPT person-trials matched to 636 non-EBP person-trials. Completing ⩾8 CPT sessions was associated with a 6.4-point greater improvement on the PCL (95% CI 3.1–10.0). There were 272 PE person-trials matched to 272 non-EBP person-trials. Completing ⩾8 PE sessions was associated with a 9.7-point greater improvement on the PCL (95% CI 5.4–13.8). There were 232 PE person-trials matched to 232 CPT person-trials. Those completing ⩾8 PE sessions had slightly greater, but not statistically significant, improvement on the PCL (8.3-points; 95% CI 5.9–10.6) than those completing ⩾8 CPT sessions (7.0-points; 95% CI 5.5–8.5).

**Conclusions:**

PTSD symptom improvement was similar and modest for both EBPs. Although EBPs are helpful, research to further improve PTSD care is critical.

## Introduction

Prolonged exposure (PE) and cognitive processing therapy (CPT) are first-line treatments for posttraumatic stress disorder (PTSD) based on clinical practice guidelines (Departments of Veterans Affairs and Defense, 2010, 2017), supported by multiple randomized controlled trials (RCTs; Haagen, Smid, Knipscheer, Kleber, & McHugh, 2015; Monson et al., 2006; Schnurr et al., 2007; Surís, Link-Malcolm, Chard, Ahn, & North, 2013). To effectively treat PTSD, the largest integrated healthcare system in the United States, the Veterans Health Administration (VHA; Oliver, 2007), began national implementation of these two evidence-based psychotherapies (EBPs) in 2005 (Department of Veterans Affairs, 2012; Karlin et al., 2010; Karlin & Cross, 2014; Office of Mental Health, 2008; Rosen, Ruzek, & Karlin, 2017). However, some studies and systematic reviews have demonstrated that veterans and military personnel may be less likely to demonstrate clinical improvement from CPT and PE, compared to civilians (Dillon, LoSavio, Henry, Murphy, & Resick, 2019; Steenkamp, Litz, Hoge, & Marmar, 2015; Straud, Siev, Messer, & Zalta, 2019). Furthermore, CPT and PE RCTs often exclude participants with common clinical presentations of comorbid substance use disorders or suicidal ideation with intent, raising concerns regarding generalizability (Ronconi, Shiner, & Watts, 2014), despite evidence that these groups may benefit from treatment (Bryan et al., 2016; Gradus, Suvak, Wisco, Marx, & Resick, 2013; Mills et al., 2012). Given that individuals included in RCTs may not reflect the veteran population seeking clinical services, it is important to better understand whether veterans who receive CPT or PE can improve in the ‘real-world’ clinical setting (Steenkamp, Litz, & Marmar, 2020).

Another important aspect of clinical effectiveness is attending to adequate doses of treatment among those receiving PTSD EBPs, which we define as eight or more sessions, based on prior work (Cully et al., 2008; Spoont, Murdoch, Hodges, & Nugent, 2010). VHA patients initiating EBPs attend about five to nine sessions (Kehle-Forbes, Meis, Spoont, & Polusny, 2016; Watts et al., 2014), indicating that some patients may drop out of treatment. While receiving any EBP can be helpful, knowing whether individuals benefit when receiving minimally adequate treatment is important to ensure appropriate comparisons when assessing clinical outcomes.

The VHA electronic health record (EHR) provides nearly 15 years of data to evaluate whether veterans with PTSD improve from EBPs, compared to veterans who do not receive these treatments. One prior barrier to examining the real-world effectiveness of these treatments is that there was not a systematic way to track the provision of EBPs nationally through the EHR for many years. Consequently, we developed a machine-learning algorithm to identify EBPs through natural language processing (NLP; Maguen et al., 2018). Due to the robust performance metrics of our algorithm, we were able to identify patients' use of EBPs across the national network. Furthermore, focusing on post-9/11 veterans allows the study of a younger group that is more likely than prior eras to have their mental health treatment records and symptom severity level readily available. A second barrier is that until recently, there was not a reliable method of conducting comparative effectiveness trials using EHR data. There is now a series of methods papers that use EHR for emulated trials (Danaei, Rodriguez, Cantero, Logan, & Hernán, 2018; Hernán & Robins, 2016)^.^ This approach uses an advanced statistical method to create a mock RCT using EHR data by mimicking a sequence of randomized trials, one trial beginning each month of the study period.

Our objective was to compare those who receive CPT individual therapy to a non-EBP individual therapy comparator group; those who receive PE to a non-EBP individual therapy comparator group; and those receiving CPT and PE individual therapy to one another, using three emulated trials. These trials address many of the prior gaps by leveraging existing resources. First, we used machine-learning to develop an algorithm that identifies EBPs in the VHA EHR. Second, we used effectiveness and comparative effectiveness methodology designed for EHR analysis.

## Methods

### Participants

We developed a retrospective cohort of veterans with PTSD who initiated VHA mental health outpatient care using three steps. First, we identified 1 149 870 post-9/11 veterans enrolled in VHA as of September 2014. Second, we identified the subset who had a post-deployment PTSD diagnosis recorded at two outpatient visits or one inpatient stay between October 2001 and September 2015 at one of 1250 VHA facilities (130 station identifiers; *n* = 308 556). Third, we restricted the cohort to the 265 566 veterans who had both an initial post-deployment mental health visit and at least one coded psychotherapy visit with PTSD as the primary or secondary diagnosis that was linked to a clinical note (necessary to confirm receipt of EBP) by June 2017. Our cohort development strategy provided a minimum of 21 months of follow-up from PTSD diagnosis to the end of study. This study was approved by the University of California, San Francisco and San Francisco VA Health Care System Institutional Review Board.

### Sources of data

The Operation Enduring Freedom (OEF)/Operation Iraqi Freedom (OIF)/Operation New Dawn (OND) Roster was used to identify post-9/11 veterans enrolled in VHA. We obtained demographic and military service information from the OEF/OIF/OND Roster and linked this information to the VHA Corporate Data Warehouse (CDW), a national repository of VHA clinical and administrative data. Diagnostic information, healthcare utilization, clinical pain scores, suicide risk screening data, and pharmacy data were retrieved from the CDW. Preliminary identification of psychotherapy visits was made using common procedural terminology codes (see online Supplement S1). Text notes linked to those visits were extracted from CDW. PTSD Checklist (PCL) scores were obtained either from CDW structured data or extracted from psychotherapy notes using an NLP algorithm (see online Supplement S2).

### Identifying evidence-based psychotherapy

Due to challenges in reliably identifying CPT and PE used in the VHA EHR (Shiner et al., 2012), we recently developed an algorithm to identify EBPs through NLP of psychotherapy notes (Maguen et al., 2018). Using this NLP system, we classified all PTSD psychotherapy notes into one of five classes: PE, CPT individual, CPT group, other/non-EBP psychotherapy, and not psychotherapy. PTSD visits were linked to 8.1 million psychotherapy notes, and annotators labeled 3467 randomly selected psychotherapy notes (*κ* = 0.88) to indicate receipt of EBP. Our overall classification accuracy was strong (e.g. 0.991 for PE; 0.965 for CPT individual, and 0.968 for CPT group; Maguen et al., 2018). We then created a dataset with one distinct type of visit per patient-date. Since patients often had more than one type of visit on a given day, we applied a hierarchy giving EBP priority over other/non-EBP, and giving other/non-EBP priority over not psychotherapy. We allowed 24 weeks to complete an EBP, to allow ample time between sessions, given our prior findings of gaps between visits (Maguen, Madden, Cohen, Bertenthal, & Seal, 2012). CPT is available as group therapy and individual therapy. We used NLP and common procedure terminology codes to distinguish groups from individual CPT.

### Outcome

Beginning in October 2007, PCL scores were recorded by clinicians using a VHA-wide tool and uploaded nightly to the CDW. We obtained all completed (i.e. valid response to all items) PCL for DSM-IV (PCL-4) and PCL for DSM-5 (PCL-5) measures stored in the CDW for our cohort. Additional PCL total scores were obtained using NLP as recorded by clinicians in free-text clinical notes using a rule-based approach and regular expressions. The NLP system was able to accurately distinguish the different versions of the PCL (for DSM-IV and DSM-5), and validation analysis suggested a precision of 98% (see online Supplement S2). We pooled the two sources of PCL score data (i.e. CDW, NLP) and removed duplicate and impossible values, resulting in 603 575 PCL-4 measurements for 112 939 patients and 74 383 PCL-5 measurements for 27 342 patients. A total of 116 539 patients had at least one PCL score, and 149 027 patients were without PCL data. For the analytical sample (see details below), we retained the PCLs measured within 14 days before or after each trial-specific start date and PCLs measured within 7 days before or after the trial end date. For patients with more than one PCL at trial-specific start or end, we kept the one absolute closest in time to start (end). Patients had to have the same PCL version recorded at the start and end of a specific trial to be included in analysis. We standardized and transformed PCL-4 and PCL-5 measures to *z*-scores so that both PCL versions could be modeled on the same scale. We calculated PCL-4 and PCL-5 total score means and standard deviations at the start of each trial, confirmed that these distributions were comparable with published trials data (Hoge, Riviere, Wilk, & Weathers, 2014; Wortmann et al., 2016), and then calculated follow-up PCL *z*-scores (primary outcome). For the presentation of results, we back-transformed *z*-scores into the PCL-4 scale since it was most common in our data. Secondary outcomes included percentage change at follow-up from the baseline PCL score, and recovery at follow-up (i.e. improvement of ⩾10-points and follow-up PCL-4 score <39; Litz et al., 2019).

### Analytical sample

We used the framework outlined and applied by Hernan et al. for comparative effectiveness research using observational data to emulate a target randomized trial (Hernán & Robins, 2016; Hernán, Sauer, Hernandez-Diaz, Platt, & Shrier, 2016). Our analyses targeted the observational analog of initiator and completer analyses, for each of three treatment comparisons: CPT individual *v.* non-EBP individual, PE *v.* non-EBP individual, and PE *v.* CPT individual psychotherapy.

Observation years with available PCL data (10/2007–6/2017) were divided into calendar months, representing 112 possible trial start dates after accounting for low data representation in the initial 3 months and cutting off the final 6 months to allow for observation of late-enrolling patients. Patients could be enrolled in as many of the trials as they were eligible, with enrollment in a trial for a unique patient representing a person-trial (Danaei, Rodriguez, Cantero, Logan, & Hernán, 2013; Hernán et al., 2008). We excluded person-trials for patients who had engaged in CPT or PE psychotherapy in the previous 30 days, those without PCL measured at the trial start date, and those without follow-up PCL at the trial end.

**Fig. 1.**
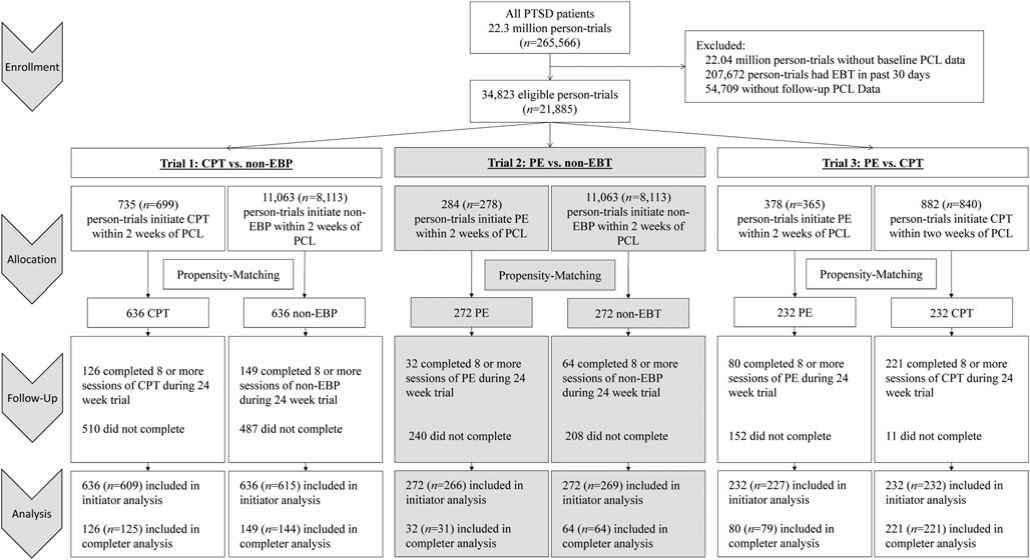
Consort diagram of sample selection showing the number of person-trials with the number of unique patients in parentheses.

Eligible person-trials were given a ‘grace period’ of 2 weeks from the start of a trial to initiate EBP and were assigned to treatment groups accordingly. Specifically, person-trials who initiated CPT or PE individual therapy were assigned to CPT or PE groups, respectively; otherwise, if they initiated non-EBP individual psychotherapy they were assigned to the non-EBP group. Completers were defined as those who completed at least eight sessions of the assigned treatment by the trial end (Department of Veterans Affairs, 2012; Maguen et al., 2019; Spoont et al., 2010). All person-trials were followed for 24 weeks (Fig. 1).

Our person-trial selection approach contained three potential sources of bias due to non-random variability in both baseline and time-variant patient characteristics (e.g. prior EBP use, medications, comorbidities). First, eligible patients with a baseline PCL measurement who are excluded due to a lack of a follow-up PCL may differ from those who have a follow-up PCL (i.e. selection bias). Second, among patients who have both a baseline and follow-up PCL, initiators of EBP may differ from initiators of non-EBP and initiators of PE may differ from initiators of CPT (i.e. confounding bias). Third, among patients who have both a baseline and follow-up PCL, completers may differ from initiators who do not complete eight sessions of treatment (i.e. non-adherence bias).

To account for selection bias due to lack of follow-up PCL, we used inverse probability weights – a form of propensity scores that allows for time-dependent confounding and are therefore more appropriate when adjusting for loss to follow-up. Among all eligible patients with a baseline PCL measure, we estimated the loss to follow-up weights with pooled logistic regression, in which the dependent variable was the indicator for follow-up PCL and independent variables included an indicator for person-trial starting month, and an anti-parsimonious specification of variables that have a plausible correlation with the outcome (see online Supplement S3, Table S3).

To account for potential baseline confounding of treatment group initiation and PTSD symptoms, we implemented propensity score matching among those with both baseline and follow-up PCL (Ross et al., 2015). We performed propensity score matching separately for each of the three treatment comparisons of interest: (1) person-trials in the non-EBP control group with person-trials in the CPT group, (2) person-trials in the non-EBP control group with person-trials in PE group, and (3) person-trials in CPT group with person-trials in PE group. We used greedy 1:1 nearest neighbor matching, with exact matching on trial, without replacement, within caliper widths <0.2 (Austin, 2014). Due to the small number of eligible persons initiating EBP within 2 weeks of the start of the trial, the number of predictors included in the logistic regression models used to create the propensity scores was necessarily restricted to baseline variables known a priori to be associated with both treatment selection and follow-up PCL score: age, baseline PCL *z*-score, and comorbid conditions (alcohol abuse, alcohol dependence, depression, drug abuse, drug dependence, psychosis, pain, suicide, and TBI; Holder et al., 2020). The EBP initiator and non-EBP initiator groups were compared with standardized mean differences, where an absolute difference of <0.1 on confounders of age and comorbidities was indicative of group similarity (e.g. good balance). A total of 636 CPT individual therapy initiators were matched to 636 non-EBP individual therapy initiator controls, 272 PE initiators were matched to 272 non-EBP initiator controls, and 232 CPT initiators were matched to 232 PE initiators (Fig. 1).

Finally, for the completer analysis, we implemented inverse probability of censoring weights (IPCWs) to account for non-adherence in both treatment groups. IPCW recreates the population that would have been observed had patients adhered to the assigned treatment strategy. We artificially censored person-trials when they deviated from completing at least eight sessions of the treatment that they initiated during the first 2 weeks of trial (i.e. assigned treatment strategy) and assigned weights that were proportional to the inverse of the probability of remaining adherent to the treatment given each individual patient characteristic (see online Supplement S4 for details of models of non-adherence bias).

Prior engagement in EBPs, psychosis, as well as several other baselines and time-updated covariates (see online Supplement Tables S3 and S4) were included in the logistic regression predicting having a follow-up PCL to estimate the inverse probability of follow-up weights (selection bias), and also in extended Cox models to estimate IPCWs (non-adherence bias).

### Statistical analysis

Baseline demographic characteristics and comorbid psychiatric conditions, in matched samples, were summarized with means and standardized deviations for continuous variables, and with frequency distributions for categorical variables (Table 1).

**Table 1.** Baseline characteristics in matched samples for each of the treatment effectiveness comparisons

	CPT *v*. non-EBP			PE *v*. non-EBP			PE *v*. CPT individual		
Variable	Non-EBP (*N* = 636)	CPT (*N* = 636)	Standardized mean difference	Non-EBP (*N* = 272)	PE (*N* = 272)	Standardized mean difference	CPT individual (*N* = 232)	PE (*N* = 232)	Standardized mean difference
Age	36.3 ± 8.9	36.7 ± 8.8	0.04	36.47 ± 8.81	36.50 ± 8.67	0.004	36.6 ± 9.1	36.7 ± 8.7	0.01
Female	71 (11.2%)	111 (17.5%)	0.18	31 (11.4%)	34 (12.5%)	0.034	172 (74.1%)	200 (86.2%)	0.32
Race (self-report)			0.14			0.18			0.17
White	489 (76.9%)	473 (74.4%)		203 (74.6%)	192 (70.6%)	203 (74.6%)	167 (72.0%)	162 (69.8%)	
Black	74 (11.6%)	89 (14.0%)		38 (14.0%)	48 (17.6%)	38 (14.0%)	37 (15.9%)	39 (16.8%)	
American Indian or Alaska Native	11 (1.7%)	7 (1.1%)		3 (1.1%)	2 (0.7%)	3 (1.1%)	3 (1.3%)	2 (0.9%)	
Asian	14 (2.2%)	10 (1.6%)		6 (2.2%)	2 (0.7%)	6 (2.2%)	4 (1.7%)	4 (1.7%)	
Native Hawaiian or other Pacific Islander	9 (1.4%)	13 (2.0%)		6 (2.2%)	3 (1.1%)	6 (2.2%)	4 (1.7%)	3 (1.3%)	
Multi-race	7 (1.1%)	9 (1.4%)		3 (1.1%)	3 (1.1%)	3 (1.1%)	4 (1.7%)	2 (0.9%)	
Unknown	32 (5.0%)	35 (5.5%)		13 (4.8%)	22 (8.1%)	13 (4.8%)	13 (5.6%)	20 (8.6%)	
Ethnicity (self-report)			0.06			0.28			0.26
Hispanic or Latino	69 (10.8%)	80 (12.6%)		29 (10.7%)	44 (16.2%)		30 (12.9%)	38 (16.4%)	
Not Hispanic or Latino	545 (85.7%)	534 (84.0%)		229 (84.2%)	225 (82.7%)		193 (83.2%)	193 (83.2%)	
Other	14 (2.2%)	15 (2.4%)		8 (2.9%)	3 (1.1%)		7 (3.0%)	1 (0.4%)	
Unknown	8 (1.3%)	7 (1.1%)		6 (2.2%)	0 (0.0%)		2 (0.9%)	0 (0.0%)	
Baseline PCL *z*-score	0.17 ± 0.90	0.14 ± 0.93	−0.03	0.18 ± 0.88	0.10 ± 0.86	−0.1	0.11 ± 0.99	0.07 ± 0.88	−0.04
Comorbidities									
Alcohol abuse	76 (11.9%)	68 (10.7%)	−0.04	44 (16.2%)	36 (13.2%)	−0.08	19 (8.2%)	19 (8.2%)	0.00
Alcohol dependence	63 (9.9%)	63 (9.9%)	0.00	34 (12.5%)	34 (12.5%)	<0.001	15 (6.5%)	18 (7.8%)	0.05
Drug abuse	28 (4.4%)	37 (5.8%)	0.06	12 (4.4%)	16 (5.9%)	0.07	4 (1.7%)	5 (2.2%)	0.03
Drug dependence	31 (4.9%)	43 (6.8%)	0.08	17 (6.3%)	18 (6.6%)	0.015	9 (3.9%)	10 (4.3%)	0.02
Psychosis	170 (26.7%)	184 (28.9%)	0.05	81 (29.8%)	83 (30.5%)	0.016	40 (17.2%)	60 (25.9%)	0.21
Pain	348 (54.7%)	332 (52.2%)	−0.05	146 (53.7%)	155 (57.0%)	0.07	121 (52.2%)	118 (50.9%)	−0.03
Suicide	29 (4.6%)	29 (4.6%)	0.00	20 (7.4%)	16 (5.9%)	−0.06	10 (4.3%)	9 (3.9%)	−0.02
TBI	88 (13.8%)	99 (15.6%)	0.05	45 (16.5%)	42 (15.4%)	−0.03	39 (16.8%)	36 (15.5%)	−0.03

#### Primary outcome analysis

We estimated the observational analog of an initiator effect with a general linear model of baseline and follow-up PCL scores, weighted to account for informative follow-up (see online Supplement S3, Table S3), with treatment group, time, and their interaction as the only predictors. We then estimated the observational analog of a completer effect. We retained patients who were adherent to the assigned treatment strategy and conducted the primary outcome analysis using a general linear model of baseline and follow-up PCL scores, weighted to account for both informative follow-up (see online Supplement S3, Table S3) and differential non-adherence (details of censoring rules and models in online Supplement S4), with treatment group, time, and their interaction as the only predictors.

#### Secondary outcomes analysis

The difference in relative change from baseline was estimated using a generalized linear model of within-person percent change relative to baseline PCL with treatment group as the main predictor and baseline PCL score as the only covariate.

The between-group difference for percent recovered (see Outcome section) was calculated with corresponding exact 95% confidence intervals (CIs). The relative risk difference for recovery was estimated by the treatment group odds ratio.

All analyses of treatment initiators were weighted to account for informative follow-up (i.e. IPCW of follow-up PCL, online Supplement S3, Table S3), and all analyses of treatment completers were weighted to account for both informative follow-up and differential non-adherence (i.e. IPCW, online Supplement Table S4). Robust 95% CIs were estimated from 500 bootstrapped samples.

#### Sensitivity analysis

We performed a sensitivity analysis with an alternate analytical approach – cloning/censoring/weighting (Danaei et al., 2018; Hernán, 2018; see online Supplement Fig. S5A). The aim of the sensitivity analysis was to estimate the average treatment effect (ATE) among the population of all eligible PTSD patients, whereas the aim of the primary analysis was to estimate the average treatment effect among the treated (ATT; i.e. those who initiated EBP). In the ideal scenario of a randomized control trial (RCT), the ATE equals ATT. Theoretically, if all potential sources of bias were perfectly accounted for, we would expect primary results to be the same as sensitivity results.

Briefly, the analytical approach for estimating ATE assigned patient replicates (clones) to treatment groups to achieve a perfect balance on confounders at baseline, allowed clones assigned to EBP to initiate treatment at any point during the 24-week ‘trial,’ censored clones when they deviated from assigned treatment (earliest of treatment switching or failure to initiate), and finally, weighted the uncensored analytical sample to reflect population of eligible patients at baseline. The analytical samples for sensitivity analyses are shown in online Supplement Fig. S5A. Baseline characteristics of the 89 532 eligible person-trials are presented in online Supplement Table S5A. The sensitivity analysis modeled the PCL outcome treatment as the main predictor, with baseline PCL as the only covariate to account for any residual confounding, and models were weighted by inverse probability of follow-up and inverse probability of censoring. All analyses were conducted using SAS Enterprise Guide version 7.2 (SAS Institute, Inc.).

## Results

### CPT *v.* non-EBP standard of care

**Fig. 2.**
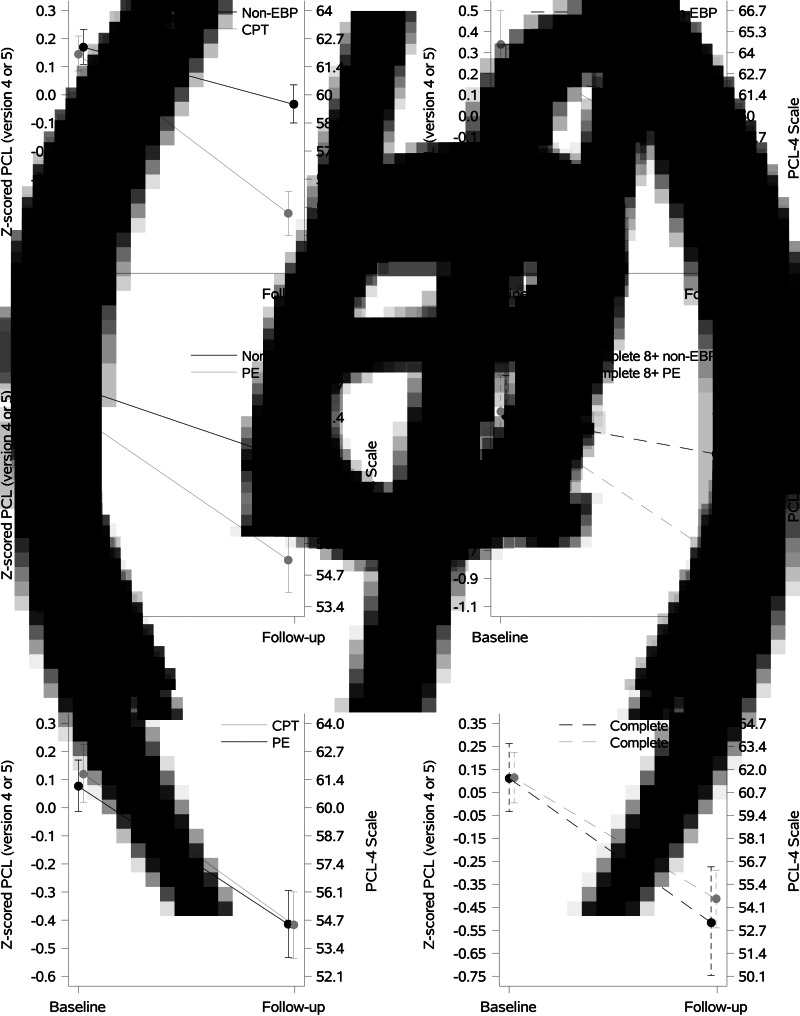
Line figures of Z-scored PCL at baseline and follow-up by treatment group. Patients who initiated EBP within 2 weeks of baseline were matched to non-EBP controls (propensity score matching) and the resulting matched sample was weighted by inverse probability of follow-up. Figures A, C and E are analogous to an ITT analysis, where patients in the treatment group may not have perfect adherence to their assigned treatment. Figure B, D and F are analogous to a complete adherence analysis, where patients who deviated from their assigned treatment strategy were artificially censored and the uncensored patients were weighted by inverse probability of censoring.

There were 636 CPT initiator person-trials matched to 636 non-EBP person-trials (Fig. 1), with very good balance (mean standardized differences <0.1) on baseline confounders, age, and comorbidities. Both the CPT and non-EBP groups experienced a reduction in PTSD symptoms by the end of the 24-week follow-up (Fig. 2). In the initiator sample, the CPT group improved by 7.5-points (95% CI 6.7–8.3) and the non-EBP group improved by 2.7-points (95% CI 2.0–3.4). CPT initiators had a 4.8-point greater improvement on the PCL-4 scale, 7.3-percentage points greater improvement relative to baseline, and 7.4% more experienced recovery (2.6-times greater odds) compared to non-EBP initiators.

In the completer sample, those completing ⩾8 CPT sessions improved by 8.2-points (95% CI 5.1–11.6) and those completing ⩾8 non-EBP sessions improved by 1.8-points (95% CI 0.6–3.1). Those completing ⩾8 sessions of CPT had a 6.4-point greater improvement on the PCL-4 scale, 17-percentage point greater improvement on the PCL relative to baseline, and 11.9% more experienced recovery (8.7-times greater odds) compared to those completing ⩾8 sessions of non-EBP (Table 2).

**Table 2.** Treatment effect of evidence-based psychotherapy for PTSD in VHA

Treatment comparison	Treatment effect in *z*-values (95% CI)	Treatment effect in PCL-4 scale	Between-group difference in percent change from baseline	Between-group difference for percent recovered^a^ (95% CI)	Odds ratio for recovery^a^ (95% CI)
CPT initiator *v*. non-EBP initiator	−0.36 (−0.44 to −0.28)	−4.8 (−5.8, 3.7)	−7.3 (−10.1, −4.5)	7.4 (5.4–9.5)	2.6 (2.0–3.4)
Complete 8 + CPT *v*. Complete 8 + non-EBP	−0.49 (−0.75 to −0.23)	−6.4 (−10.0, −3.1)	−17.0 (−22.9, −11.1)	11.9 (7.7–16.2)	8.7 (3.7–20.8)
PE initiator *v*. Non-EBP initiator	−0.23 (−0.36 to −0.10)	−3.0 (−4.8, −1.3)	−5.2 (−9.0, −1.4)	5.5 (2.5–8.5)	2.2 (1.4–3.3)
Complete PE 8 + Complete 8 + non-EBP	−0.73 (−1.04 to −0.41)	−9.7 (−13.8, −5.4)	−14.5 (−21.5, −7.6)	2.8 (−2.1–7.6)	1.9 (0.6–6.2)
PE initiator *v*. CPT initiator	0.05 (−0.11 to 0.22)	0.6 (−1.5, 2.9)	0.4 (−4.1, 4.8)	1.8 (−2.1–5.8)	1.2 (0.8–1.8)
Complete 8 + PE Complete 8 + CPT	−0.10 (−0.32 to 0.11)	−1.3 (−4.2, 1.4)	−4.1 (−11.4, 3.3)	5.1 (−3.0–13.2)	1.6 (0.8–3.0)

a Recovery defined as having experienced a 10-point or greater improvement from baseline and follow-up PCL-4 score <39.

^b^Effect is the difference between propensity-score matched treatment and control group in the change in *z*-scored PCL (i.e. difference-in-difference), weighted by inverse probability of having follow-up PCL. The complete adherence effect additionally accounted for treatment switching or lack of adherence with inverse probability of censoring weights. A negative value means the treatment group had greater improvement (decrease) in symptoms than the control group, e.g. CPT completers had a 0.49 greater decrease in *z*-score (or, 6.4-points on the PCL-4 scale) compared to the non-EBP group. *Note*. Initiators are those receiving any individual therapy and completers are those receiving eight or more individual therapy sessions.

### PE *v.* non-EBP standard of care

There were 272 PE person-trials matched to 272 non-EBP person-trials (Fig. 1), with very good balance (mean standardized differences <0.1) on baseline confounders, age, and comorbidities (Table 1). Both the PE and non-EBP groups experienced a reduction in PTSD symptoms by the end of the 24-week follow-up (Fig. 2). In the initiator sample, the PE group had a 3.0-point greater improvement (on PCL-4 scale) compared to non-EBP (Table 2). Relative to baseline, initiators of PE improved by 5.2-percentage points more on PCL, and 5.5% more PE initiators experienced recovery (2.2-fold greater odds) compared to non-EBP initiators.

Those completing ⩾8 PE sessions improved by 13.3-points (95% CI 9.5–17.0) and those completing ⩾8 non-EBP sessions improved by 3.6-points (95% CI 1.8–5.5). In the completer sample, the group that completed ⩾8 sessions of PE had a 9.7-point greater improvement on the PCL-4 scale (95% CI 5.4–13.8), relative change from baseline was 14.5-percentage points greater, and 2.8% more PE completers experiencing recovery (1.9 times greater odds) compared to non-EBP completers (Table 2).

### PE *v.* CPT individual

There were 232 PE initiator person-trials matched to 232 CPT individual initiator person-trials (Fig. 1), with a very good balance on all baseline confounders (mean standardized differences <0.01, except psychosis). The absolute level of improvement on PCL scores, the relative change in PCL score from baseline, and the rate of recovery did not differ in PE and CPT initiator groups (Table 2).

Those completing ⩾8 PE sessions improved by 8.3-points (95% CI 5.9–10.6) and those completing ⩾8 CPT sessions improved by 7.0-points (95% CI 5.5–8.5) (Fig. 2). Those completing ⩾8 PE sessions had a similar level of improvement in PCL score compared to those completing ⩾8 CPT. Relative change from baseline and rate of recovery was greater in those completing ⩾8 PE sessions compared to those completing ⩾8 CPT sessions; however, differences were not statistically significant.

### Sensitivity analyses

We found similar results in our sensitivity analyses aimed at the average treatment effect (online Supplement S5). Results of the sensitivity analyses are qualitatively similar to the primary analysis, with EBP completer groups experiencing greater improvement in PCL score compared to non-EBP completers, and PE completers experiencing slightly greater (3-points on PCL-4 scale) improvement compared to CPT completers (online Supplementary Table S5B).

## Discussion

We were able to conduct three emulated trials of PTSD EBP over a nearly 15-year period with post-9/11 veterans who sought care at the VHA. We found that while patients engaging in EBPs experienced some improvement compared to controls, the improvement did not reach levels observed in EBP RCTs (Haagen et al., 2015; Monson et al., 2006; Schnurr et al., 2007; Surís et al., 2013). While improvement was statistically significant when comparing CPT to non-EBP and PE to non-EBP, overall levels of improvement remained modest. One possible reason for this discrepancy with clinical trials is that patients in routine clinical practice are more variable in their engagement, ambivalence, motivation, and skepticism of treatment, compared to those enrolled in RCTs (Hundt et al., 2015). Second, many veterans with elevated acute risk for suicide or substance use disorders are excluded from RCTs (Ronconi et al., 2014). Third, there may be differences in treatment delivery between providers in clinical practice and trial therapists, which may affect outcomes (Holder, Holliday, Williams, Mullen, & Surís, 2018). Treatment fidelity is closely monitored in RCTs and there may be more variation or modifications to EBPs that are provided in clinical practice (Thompson, Simiola, Schnurr, Stirman, & Cook, 2018), where typically fidelity is not as closely monitored. Our control group, as well as the individuals in our study, reflected real-world treatment. While these patients did not receive either CPT or PE, they may have received other helpful individual therapies available in mental health clinics, although overall treatment gains were small compared to EBPs. This is in contrast to control groups in RCTs, some of which are limited to the attention or wait-list control group.

We were also able to compare CPT and PE and found that there were no clinically significant differences between these two groups, consistent with one prior RCT among civilians (Resick, Nishith, Weaver, Astin, & Feuer, 2002). This confirms that either treatment can be beneficial, patient preferences should be taken into account, and shared decision making can guide treatment planning. Given that dropout rates from these treatments are sizeable, prior research shedding light on which patients are most likely to complete a particular EBP should be considered. For example, we previously found that individuals with a history of suicidal ideation or attempts were more likely to complete PE, but not CPT (Maguen et al., 2019).

One of the notable benefits of doing these emulated trials in a real-world clinic setting is that veterans with multiple comorbid disorders are included in our analyses. For example, a significant percentage of veterans who seek care and receive EBPs in the VA have comorbidities like traumatic brain injury (TBI), which may make it more challenging to engage, complete, and benefit from EBPs; however, this study demonstrates that even those with PTSD and comorbid mental health disorders can benefit from EBPs.

There are several limitations that should be considered when interpreting these findings. First, our study included post-9/11 veterans who sought VHA care. Consequently, these results may not generalize to older veterans or veterans of different eras, and age has been found to predict improvement from EBPs (Rizvi, Vogt, & Resick, 2009). Furthermore, these findings may not generalize to veterans who seek care outside of VHA. We also used procedure codes coupled with an NLP algorithm to identify instances of EBPs. Although the algorithm's performance was strong, there may have been a few instances of these therapies that were categorized incorrectly. Another limitation was that not all veterans receiving PTSD care receive measures tracking their symptoms and therefore may have been left out of our sample. Although the VHA has initiatives that emphasize the importance of measurement-based care, these data were collected over many years and there may be heterogeneity in symptom assessment during PE and CPT delivery across the implementation period. Finally, many PTSD symptom measures were in the clinical notes rather than in an easily extractable structured format, and although we were able to get an additional 28% of measurements through an NLP algorithm we developed, there may be errors associated with PCL extraction, despite steps to prevent this (e.g. excluding impossible values). There were also two versions of the PCL that were used during the study period, and although we used *z*-scores and were able to back transform values, errors are possible with multiple measure versions. Finally, although PCL is used to track EBP improvement throughout the VA system and across national clinics, reflecting current measurement-based practices with EBPs (Peterson, Anderson, & Bourne, 2018), it is not a clinician-administered clinical interview, which should be noted.

Despite these limitations, our results shed light on the clinical effectiveness of CPT and PE in real-world settings, compared to controls and one another. Conducting three trials in real-world settings would have been cost prohibitive if not emulated through retrospective EHR data analysis. The information gleaned from these data has never before been available due to data extraction and methods limitations. Importantly, by leveraging these methods we found that the magnitude of benefit from CPT and PE in clinical practice is lower than expected in RCTs. This may also partially explain poor national uptake. It is critical to better understand this discrepancy, and whether it is due to patient ambivalence about these treatments, external validity (i.e. excluding clinically complex patients from RCTs), or differences in fidelity to the protocol. Furthermore, these findings can be used to provide realistic expectations for patients' levels of improvement from these treatments as well as providing a better understanding that even in real-world settings, both EBPs are equally effective, leading clinicians to lean more on shared decision making when discussing treatment planning with patients with PTSD.

Although CPT and PE are both used across the VA system, our findings demonstrate that although veterans improve, the degree of improvement is minimal and significant PTSD symptoms remain even after EBPs. Although there is room for improvement, EBPs are better than non-EBP psychotherapies provided. Consequently, it is important to acknowledge that although EBPs are helpful, research to further improve PTSD care is critical. The results underscore the need to develop better therapies or refine existing treatments for PTSD to provide more robust symptom relief.

## Supplementary material

For supplementary material accompanying this paper visit https://doi.org/10.1017/S0033291721001628. click here to view supplementary material
