# Supplementary material for: Effectiveness and comparative effectiveness of evidence-based psychotherapies for posttraumatic stress disorder in clinical practice
Source: Psychol Med. 2021 May 18;53(2):419–28. doi: 10.1017/S0033291721001628 (PMC9899565; doi:10.1017/S0033291721001628)
Supplement: Supplementary file 1 [file S0033291721001628sup001.docx]

**Supplementary Material:**

**S1. Preliminary Identification of Psychotherapy Visits Using Common Procedural Terminology Codes**

| Code | Description |
| --- | --- |
| 4062F | PATIENT REFERRAL FOR PSYCHOTHERAPY DOCUMENTED (MDD, MDD ADOL) |
| 4064F | ANTIDEPRESSANT PHARMACOTHERAPY PRESCRIBED (MDD, MDD ADOL) |
| 4065F | ANTIPSYCHOTIC PHARMACOTHERAPY PRESCRIBED (MDD) |
| 90804 | INDIVIDUAL PSYCHOTHERAPY, INSIGHT ORIENTED, BEHAVIOR MODIFYING AND/OR SUPPORTIVE, IN AN OFFICE OR OUTPATIENT FACILITY, APPROXIMATELY 20 TO 30 MINUTES FACE-TO-FACE WITH THE PATIENT; |
| 90805 | INDIVIDUAL PSYCHOTHERAPY, INSIGHT ORIENTED, BEHAVIOR MODIFYING AND/OR SUPPORTIVE, IN AN OFFICE OR OUTPATIENT FACILITY, APPROXIMATELY 20 TO 30 MINUTES FACE-TO-FACE WITH THE PATIENT; WITH MEDICAL EVALUATION AND MANAGEMENT SERVICES |
| 90806 | INDIVIDUAL PSYCHOTHERAPY, INSIGHT ORIENTED, BEHAVIOR MODIFYING AND/OR SUPPORTIVE, IN AN OFFICE OR OUTPATIENT FACILITY, APPROXIMATELY 45 TO 50 MINUTES FACE-TO-FACE WITH THE PATIENT; |
| 90807 | INDIVIDUAL PSYCHOTHERAPY, INSIGHT ORIENTED, BEHAVIOR MODIFYING AND/OR SUPPORTIVE, IN AN OFFICE OR OUTPATIENT FACILITY, APPROXIMATELY 45 TO 50 MINUTES FACE-TO-FACE WITH THE PATIENT; WITH MEDICAL EVALUATION AND MANAGEMENT SERVICES |
| 90808 | INDIVIDUAL PSYCHOTHERAPY, INSIGHT ORIENTED, BEHAVIOR MODIFYING AND/OR SUPPORTIVE, IN AN OFFICE OR OUTPATIENT FACILITY, APPROXIMATELY 75 TO 80 MINUTES FACE-TO-FACE WITH THE PATIENT; |
| 90809 | INDIVIDUAL PSYCHOTHERAPY, INSIGHT ORIENTED, BEHAVIOR MODIFYING AND/OR SUPPORTIVE, IN AN OFFICE OR OUTPATIENT FACILITY, APPROXIMATELY 75 TO 80 MINUTES FACE-TO-FACE WITH THE PATIENT; WITH MEDICAL EVALUATION AND MANAGEMENT SERVICES |
| 90810 | INDIVIDUAL PSYCHOTHERAPY, INTERACTIVE, USING PLAY EQUIPMENT, PHYSICAL DEVICES, LANGUAGE INTERPRETER, OR OTHER MECHANISMS OF NON-VERBAL COMMUNICATION, IN AN OFFICE OR OUTPATIENT FACILITY, APPROXIMATELY 20 TO 30 MINUTES FACE-TO-FACE WITH THE PATIENT; |
| 90811 | INDIVIDUAL PSYCHOTHERAPY, INTERACTIVE, USING PLAY EQUIPMENT, PHYSICAL DEVICES, LANGUAGE INTERPRETER, OR OTHER MECHANISMS OF NON-VERBAL COMMUNICATION, IN AN OFFICE OR OUTPATIENT FACILITY, APPROXIMATELY 20 TO 30 MINUTES FACE-TO-FACE WITH THE PATIENT; WITH MEDICAL EVALUATION AND MANAGEMENT SERVICES |
| 90812 | INDIVIDUAL PSYCHOTHERAPY, INTERACTIVE, USING PLAY EQUIPMENT, PHYSICAL DEVICES, LANGUAGE INTERPRETER, OR OTHER MECHANISMS OF NON-VERBAL COMMUNICATION, IN AN OFFICE OR OUTPATIENT FACILITY, APPROXIMATELY 45 TO 50 MINUTES FACE-TO-FACE WITH THE PATIENT; |
| 90813 | INDIVIDUAL PSYCHOTHERAPY, INTERACTIVE, USING PLAY EQUIPMENT, PHYSICAL DEVICES, LANGUAGE INTERPRETER, OR OTHER MECHANISMS OF NON-VERBAL COMMUNICATION, IN AN OFFICE OR OUTPATIENT FACILITY, APPROXIMATELY 45 TO 50 MINUTES FACE-TO-FACE WITH THE PATIENT; WITH MEDICAL EVALUATION AND MANAGEMENT SERVICES |
| 90814 | INDIVIDUAL PSYCHOTHERAPY, INTERACTIVE, USING PLAY EQUIPMENT, PHYSICAL DEVICES, LANGUAGE INTERPRETER, OR OTHER MECHANISMS OF NON-VERBAL COMMUNICATION, IN AN OFFICE OR OUTPATIENT FACILITY, APPROXIMATELY 75 TO 80 MINUTES FACE-TO-FACE WITH THE PATIENT; |
| 90815 | INDIVIDUAL PSYCHOTHERAPY, INTERACTIVE, USING PLAY EQUIPMENT, PHYSICAL DEVICES, LANGUAGE INTERPRETER, OR OTHER MECHANISMS OF NON-VERBAL COMMUNICATION, IN AN OFFICE OR OUTPATIENT FACILITY, APPROXIMATELY 75 TO 80 MINUTES FACE-TO-FACE WITH THE PATIENT; WITH MEDICAL EVALUATION AND MANAGEMENT SERVICES |
| 90816 | INDIVIDUAL PSYCHOTHERAPY, INSIGHT ORIENTED, BEHAVIOR MODIFYING AND/OR SUPPORTIVE, IN AN INPATIENT HOSPITAL, PARTIAL HOSPITAL OR RESIDENTIAL CARE SETTING, APPROXIMATELY 20 TO 30 MINUTES FACE-TO-FACE WITH THE PATIENT; |
| 90817 | INDIVIDUAL PSYCHOTHERAPY, INSIGHT ORIENTED, BEHAVIOR MODIFYING AND/OR SUPPORTIVE, IN AN INPATIENT HOSPITAL, PARTIAL HOSPITAL OR RESIDENTIAL CARE SETTING, APPROXIMATELY 20 TO 30 MINUTES FACE-TO-FACE WITH THE PATIENT; WITH MEDICAL EVALUATION AND MANAGEMENT SERVICES |
| 90818 | INDIVIDUAL PSYCHOTHERAPY, INSIGHT ORIENTED, BEHAVIOR MODIFYING AND/OR SUPPORTIVE, IN AN INPATIENT HOSPITAL, PARTIAL HOSPITAL OR RESIDENTIAL CARE SETTING, APPROXIMATELY 45 TO 50 MINUTES FACE-TO-FACE WITH THE PATIENT; |
| 90819 | INDIVIDUAL PSYCHOTHERAPY, INSIGHT ORIENTED, BEHAVIOR MODIFYING AND/OR SUPPORTIVE, IN AN INPATIENT HOSPITAL, PARTIAL HOSPITAL OR RESIDENTIAL CARE SETTING, APPROXIMATELY 45 TO 50 MINUTES FACE-TO-FACE WITH THE PATIENT; WITH MEDICAL EVALUATION AND MANAGEMENT SERVICES |
| 90821 | INDIVIDUAL PSYCHOTHERAPY, INSIGHT ORIENTED, BEHAVIOR MODIFYING AND/OR SUPPORTIVE, IN AN INPATIENT HOSPITAL, PARTIAL HOSPITAL OR RESIDENTIAL CARE SETTING, APPROXIMATELY 75 TO 80 MINUTES FACE-TO-FACE WITH THE PATIENT; |
| 90822 | INDIVIDUAL PSYCHOTHERAPY, INSIGHT ORIENTED, BEHAVIOR MODIFYING AND/OR SUPPORTIVE, IN AN INPATIENT HOSPITAL, PARTIAL HOSPITAL OR RESIDENTIAL CARE SETTING, APPROXIMATELY 75 TO 80 MINUTES FACE-TO-FACE WITH THE PATIENT; WITH MEDICAL EVALUATION AND MANAGEMENT SERVICES |
| 90823 | INDIVIDUAL PSYCHOTHERAPY, INTERACTIVE, USING PLAY EQUIPMENT, PHYSICAL DEVICES, LANGUAGE INTERPRETER, OR OTHER MECHANISMS OF NON-VERBAL COMMUNICATION, IN AN INPATIENT HOSPITAL, PARTIAL HOSPITAL OR RESIDENTIAL CARE SETTING, APPROXIMATELY 20 TO 30 MINUTES FACE-TO-FACE WITH THE PATIENT; |
| 90824 | INDIVIDUAL PSYCHOTHERAPY, INTERACTIVE, USING PLAY EQUIPMENT, PHYSICAL DEVICES, LANGUAGE INTERPRETER, OR OTHER MECHANISMS OF NON-VERBAL COMMUNICATION, IN AN INPATIENT HOSPITAL, PARTIAL HOSPITAL OR RESIDENTIAL CARE SETTING, APPROXIMATELY 20 TO 30 MINUTES FACE-TO-FACE WITH THE PATIENT; WITH MEDICAL EVALUATION AND MANAGEMENT SERVICES |
| 90826 | INDIVIDUAL PSYCHOTHERAPY, INTERACTIVE, USING PLAY EQUIPMENT, PHYSICAL DEVICES, LANGUAGE INTERPRETER, OR OTHER MECHANISMS OF NON-VERBAL COMMUNICATION, IN AN INPATIENT HOSPITAL, PARTIAL HOSPITAL OR RESIDENTIAL CARE SETTING, APPROXIMATELY 45 TO 50 MINUTES FACE-TO-FACE WITH THE PATIENT; |
| 90827 | INDIVIDUAL PSYCHOTHERAPY, INTERACTIVE, USING PLAY EQUIPMENT, PHYSICAL DEVICES, LANGUAGE INTERPRETER, OR OTHER MECHANISMS OF NON-VERBAL COMMUNICATION, IN AN INPATIENT HOSPITAL, PARTIAL HOSPITAL OR RESIDENTIAL CARE SETTING, APPROXIMATELY 45 TO 50 MINUTES FACE-TO-FACE WITH THE PATIENT; WITH MEDICAL EVALUATION AND MANAGEMENT SERVICES |
| 90828 | INDIVIDUAL PSYCHOTHERAPY, INTERACTIVE, USING PLAY EQUIPMENT, PHYSICAL DEVICES, LANGUAGE INTERPRETER, OR OTHER MECHANISMS OF NON-VERBAL COMMUNICATION, IN AN INPATIENT HOSPITAL, PARTIAL HOSPITAL OR RESIDENTIAL CARE SETTING, APPROXIMATELY 75 TO 80 MINUTES FACE-TO-FACE WITH THE PATIENT; |
| 90829 | INDIVIDUAL PSYCHOTHERAPY, INTERACTIVE, USING PLAY EQUIPMENT, PHYSICAL DEVICES, LANGUAGE INTERPRETER, OR OTHER MECHANISMS OF NON-VERBAL COMMUNICATION, IN AN INPATIENT HOSPITAL, PARTIAL HOSPITAL OR RESIDENTIAL CARE SETTING, APPROXIMATELY 75 TO 80 MINUTES FACE-TO-FACE WITH THE PATIENT; WITH MEDICAL EVALUATION AND MANAGEMENT SERVICES |
| 90832 | PSYCHOTHERAPY, 30 MINUTES WITH PATIENT AND/OR FAMILY MEMBER |
| 90833 | PSYCHOTHERAPY, 30 MINUTES WITH PATIENT AND/OR FAMILY MEMBER WHEN PERFORMED WITH AN EVALUATION AND MANAGEMENT SERVICE (LIST SEPARATELY IN ADDITION TO THE CODE FOR PRIMARY PROCEDURE) |
| 90834 | PSYCHOTHERAPY, 45 MINUTES WITH PATIENT AND/OR FAMILY MEMBER |
| 90836 | PSYCHOTHERAPY, 45 MINUTES WITH PATIENT AND/OR FAMILY MEMBER WHEN PERFORMED WITH AN EVALUATION AND MANAGEMENT SERVICE (LIST SEPARATELY IN ADDITION TO THE CODE FOR PRIMARY PROCEDURE) |
| 90837 | PSYCHOTHERAPY, 60 MINUTES WITH PATIENT AND/OR FAMILY MEMBER |
| 90838 | PSYCHOTHERAPY, 60 MINUTES WITH PATIENT AND/OR FAMILY MEMBER WHEN PERFORMED WITH AN EVALUATION AND MANAGEMENT SERVICE (LIST SEPARATELY IN ADDITION TO THE CODE FOR PRIMARY PROCEDURE) |
| 90841 | INDIVIDUAL MEDICAL PSYCHOTHERAPY BY A PHYSICIAN, WITH CONTINUING MEDICAL DIAGNOSTIC EVALUATION, AND DRUG MANAGEMENT WHEN INDICATED, INCLUDING INSIGHT ORIENTED, BEHAVIOR MODIFYING OR SUPPORTIVE PSYCHOTHERAPY (FACE-TO-FACE WITH THE PATIENT); TIME UNSPECIFIED |
| 90843 | INDIVIDUAL MEDICAL PSYCHOTHERAPY BY A PHYSICIAN, WITH CONTINUING MEDICAL DIAGNOSTIC EVALUATION, AND DRUG MANAGEMENT WHEN INDICATED, INCLUDING INSIGHT ORIENTED, BEHAVIOR MODIFYING OR SUPPORTIVE PSYCHOTHERAPY (FACE-TO-FACE WITH THE PATIENT); APPROXIMATELY 20 TO 30 MINUTES |
| 90844 | INDIVIDUAL MEDICAL PSYCHOTHERAPY BY A PHYSICIAN, WITH CONTINUING MEDICAL DIAGNOSTIC EVALUATION, AND DRUG MANAGEMENT WHEN INDICATED, INCLUDING INSIGHT ORIENTED, BEHAVIOR MODIFYING OR SUPPORTIVE PSYCHOTHERAPY (FACE-TO-FACE WITH THE PATIENT); APPROXIMATELY 45 TO 50 MINUTES |
| 90853 | GROUP PSYCHOTHERAPY (OTHER THAN OF A MULTIPLE-FAMILY GROUP) |
| 90855 | INTERACTIVE INDIVIDUAL MEDICAL PSYCHOTHERAPY |
| 90857 | INTERACTIVE GROUP PSYCHOTHERAPY |
| 90862 | PHARMACOLOGIC MANAGEMENT, INCLUDING PRESCRIPTION, USE, AND REVIEW OF MEDICATION WITH NO MORE THAN MINIMAL MEDICAL PSYCHOTHERAPY |
| 90863 | PHARMACOLOGIC MANAGEMENT, INCLUDING PRESCRIPTION AND REVIEW OF MEDICATION, WHEN PERFORMED WITH PSYCHOTHERAPY SERVICES (LIST SEPARATELY IN ADDITION TO THE CODE FOR PRIMARY PROCEDURE) |

**S2. Natural Language Processing Extraction of PCL Scores**

The posttraumatic stress disorder (PTSD) checklist (PCL) is a self-report PTSD symptom severity measure that can provide a baseline and ongoing measurement of symptom improvement during the course of evidence-based psychotherapy (EBP). While some PCL scores in the electronic health record (EHR) are in a coded format that can be easily extracted for analysis, we determined that a sizeable percentage (28%) were recorded only in clinical notes and were consequently inaccessible without natural language processing (NLP). Analysis through annotation of a reference set suggested that there was some level of consistency in how PCL name-value pairs are expressed in the notes, which indicated feasibility of developing an NLP system for value extraction. A rule-based NLP system was created to identify mentions of PCL score in clinical notes and to extract the specific version of the questionnaire (PCL-4, PCL-5, PCL-M, PCL-S, PCL-C) and the associated numeric score. The system was built using ﻿Leo framework based on Unstructured Information Management Architecture Asynchronous Scaleout (UIMA AS) and incorporated regular expressions and a set of patterns to find mentions of PCL results in notes (Cornia et al., 2014; Ferrucci & Lally, 2004). Once the system was developed, all psychotherapy notes in our corpus were processed, and 200 notes with a least one NLP-identified PCL value were manually confirmed by clinical chart annotators. Validation analysis suggested that the NLP system achieved precision of 98%. The NLP system was able to accurately identify mentions of PCL scores and distinguish the different versions of the PCL (for DSM-IV and DSM-5).

**S3. Accounting for Selection Bias using Inverse Probability of Having a Follow-Up PCL Measure**

We used inverse probability weighting (IPW) to deal with the possible bias due to the requirement of having a PCL measurement at the end of the trial (i.e., selection bias). To estimate the weights, patients who met all eligibility criteria at beginning of trial were included in a logistic regression model of having a final PCL measurement. The model included both baseline and post-baseline covariates (Table S3A). We computed the inverse probability weight for each person as 1 divided by the predicted value from the logistic regression model.

Table S3. Covariates Included in the Logistic Regression Model Predicting Having a Follow-Up PCL

|  |
| --- |
| **Sociodemographic data** |
| 1. Age (years) |
| 2. Male |
| 3. Racial Category |
| White |
| Black |
| American Indian or Alaska Native |
| Asian |
| Native Hawaiian or other Pacific Islander |
| Multi-racial |
| Unknown |
| 4. Ethnicity |
| Hispanic or Latino |
| Not Hispanic or Latino |
| Other |
| Unknown |
| 5. Education |
| <HS Diploma |
| HS Diploma |
| >HS Diploma |
| Missing |
| 6. Marital Status |
| Married |
| Never Married |
| Divorced, Separated, or Single |
| Widowed, Widower |
| Unknown |
| **Military Service Data** |
| 7. End of Last Deployment Date |
| 8. Branch of Service |
| Air Force |
| Army |
| Coast Guard |
| Marine |
| Navy |
| 9. Military Component |
| Active Duty |
| Guard |
| Reserve |
| 10. Military Rank |
| Enlisted |
| Officer |
| Warrant |
| 11. Number of Deployments |
| Single |
| Multiple |
| Missing |
| **VA Utilization Data** |
|  |
| 12. Percent Service Connection |
| 0% |
| 1-30% |
| 30-70% |
| 70-100% |
| 13. Drive Time to Nearest Veterans Affairs Facility |
| 0-1.78hr |
| 1.79-4.14hr |
| 4.15-5.38hr |
| 5.39-9.34hr |
| Unknown |
| **Prior Psychotherapy for PTSD** |
| 14. Non-EBP/Other Psychotherapy (Sessions) |
| None |
| One |
| Two or more |
| 15. Cognitive Processing Therapy CPT (>30 days prior to start) |
| None |
| One |
| Two or more |
| 16. Prolonged Exposure Therapy PE (>30 days prior to start) |
| None |
| One |
| Two or more |
| **Acute care service use in past year (yes/no)** |
| 17. Non-Psychiatric Hospital Admission |
| 18. Residential Mental Health |
| 19. Residential Substance Abuse |
| 20. Residential Rehabilitation Treatment Program for PTSD Admission |
| 21. Acute Mental Health Admission |
| 22. Emergency Room Visits |
| **Comorbid conditions** |
| 23. Alcohol Abuse |
| 24. Alcohol Dependence |
| 25. Depression |
| 26. Drug Abuse |
| 27. Drug Dependence |
| 28. Psychosis |
| 29. Pain |
| 30. Suicidal Ideation/Attempt |
| 31. Traumatic Brain Injury |
| 32. PCL score at baseline (z-score) |
| 33. Any of Following Medications (past 90 days): |
| Mirtazapine |
| Prazosin |
| Fluoxetine |
| Citalopram |
| Sertraline |
| Amitriptyline |
| Venlafaxine |
| Paroxetine |
| Duloxetine |
| Escitalopram |
| Imipramine |
| Fluvoxamine |
| Desvenlafaxine |
| Amitriptyline-Perphenazine |
| **Post-Baseline Covariates** |
| 34. Non-EBP/Other Psychotherapy |
| None |
| One |
| Two or more |
| 35. PE or CPT |
| None |
| One |
| Two or more |
| 36. Any Post-Baseline Medication Use (yes/no) |
| 37. Any Comorbid Condition (list above) Recorded Post-Baseline (yes/no)  38. Any Non-Psychiatric Hospital Admission  39. Any Acute Mental Health Admission  40. Any Emergency Room Visit |

**S4.** **Accounting for non-Adherence Bias using Inverse Probability of Censoring Weights (IPCW)**

To estimate the analogue of per-protocol completer effect, we artificially censored person-trials when they deviated from completing at least eight sessions of the treatment that they initiated during the first two weeks of trial (i.e., assigned treatment strategy). If a person-trial was assigned to CPT (PE) treatment they were artificially censored at earliest of a) switching to PE (CPT), or b) switching to non-EBP individual psychotherapy, or c) when too few days remained in trial to complete all eight EBP sessions. If a person-trial was assigned to non-EBP they were artificially censored at earliest of a) switching to EBP, or b) when too few days remained in trial to complete all eight non-EBP sessions. Since treatment switching is likely related to PTSD symptoms (outcome of interest) and post-baseline covariates, it introduces a bias known as dependent censoring. The inverse probability censoring weight (IPCW) is used to correct for censored subjects by giving extra weight to subjects who are not censored. In this way, the PCL outcome model to estimate the treatment effect is fit as if censoring was absent.

We estimated stabilized IPCW with extended Cox models (Willems et al., 2018). Briefly, we used the Cox proportional hazards estimator for time to artificial censoring for each subject at each time point, first without covariates and then with covariates (both baseline and time-updated; See Table S4). Then the stabilized IPCW weights were calculated by dividing the unadjusted probabilities by the adjusted probabilities. Finally, we estimated the effect of completing treatment in the absence of treatment switching by using the stabilized IPCW weights.

The stabilized IPCW weights are created by modeling the censoring process separately for each treatment group. For example, in the comparison of PE with non-EBP the stabilized IPCW are estimated among the person-trials in the PE group by running an extended Cox model without covariates, and an extended Cox model with covariates (Table S4), and then dividing the unadjusted probabilities by the adjusted probabilities. Separately, those steps are repeated among the propensity-matched non-EBP group. Finally, we retained uncensored patients and conducted the outcome analysis to estimate the treatment completer effect. We used a general linear model of baseline and follow-up PCL scores, weighted to account for selection bias and informative censoring, with treatment group, time, and their interaction as the only predictors.

Table S4. Baseline and Time-Updated Covariates Included in the Extended Cox Models to Estimate IPCW weights*

|  | | |
| --- | --- | --- |
| **Sociodemographic data** | |  |
| 1. Age | |  |
| 2. Male | |  |
| 3. Racial Category | |  |
| White | |  |
| Black | |  |
| American Indian or Alaska Native | |  |
| Asian | |  |
| Native Hawaiian or other Pacific Islander | |  |
| Multi-racial | |  |
| Unknown | |  |
| 4. Ethnicity | |  |
| Hispanic or Latino | |  |
| Not Hispanic or Latino | |  |
| Other | |  |
| Unknown | |  |
| 5. Education | |  |
| <HS Diploma | |  |
| HS Diploma | |  |
| >HS Diploma | |  |
| Missing | |  |
| 6. Marital Status | |  |
| Married | |  |
| Never Married | |  |
| Divorced, Separated, or Single | |  |
| Widowed, Widower | |  |
| Unknown | |  |
| **Military Service Data** | |  |
| 7. End of Last Deployment Date | |  |
| 8. Branch of Service | |  |
| Air Force | |  |
| Army | |  |
| Coast Guard | |  |
| Marine | |  |
| Navy | |  |
| 9. Military Component | |  |
| Active Duty | |  |
| Guard | |  |
| Reserve | |  |
| 10. Military Rank | |  |
| Enlisted | |  |
| Officer | |  |
| Warrant | |  |
| 11. Number of Deployments | |  |
| Single | |  |
| Multiple | |  |
| Missing | |  |
| **VA Utilization Data** |  |  |
| 12. Percent Service Connection | |  |
| 0% | |  |
| 1-30% | |  |
| 30-70% | |  |
| 70-100% | |  |
| 13. Drive Time to Nearest Veterans Affairs Facility | |  |
| 0-1.78hr | |  |
| 1.79-4.14hr | |  |
| 4.15-5.38hr | |  |
| 5.39-9.34hr | |  |
| Unknown | |  |
| **Prior History of Psychotherapy for PTSD** | |  |
| 14. Non-EBP/Other Psychotherapy (Sessions) | |  |
| None | |  |
| One | |  |
| Two or more | |  |
| 15. Cognitive Processing Therapy CPT (>30 days prior to start) | |  |
| None | |  |
| One | |  |
| Two or more | |  |
| 16. Prolonged Exposure Therapy PE (>30 days prior to start) | |  |
| None | |  |
| One | |  |
| Two or more | |  |
| **Time-updated (cumulative) Acute Care Service Use** | |  |
| 17. Residential Mental Health | |  |
| 18. Residential Substance Abuse | |  |
| 19. Residential Rehabilitation Treatment Program for PTSD Admission | |  |
| 20. Acute Mental Health Admission | |  |
| 21. PCL Score (z-score) | |  |
| 22. Time Updated Medications Use (yes/no) | |  |
| **Time-updated (cumulative) non-Psychiatric Acute Care Service Use** | |  |
| 23. Non-Psychiatric Hospital Admission | |  |
| 24. Emergency Room Visits | |  |
| **Time Updated Comorbid Conditions** | |  |
| 25. Alcohol Abuse | |  |
| 26. Alcohol Dependence | |  |
| 27. Depression | |  |
| 28. Drug Abuse | |  |
| 29. Drug Dependence | |  |
| 30. Psychosis | |  |
| 31. Pain | |  |
| 32. Suicidal Ideation/Attempt | |  |
| 33. Traumatic Brain Injury | |  |
| 34. Time-updated Pain Score | |  |
| 35. Time updated Suicidal Risk Screening Results | |  |

*Each extended Cox model contained slightly different post-baseline time-updated covariates when covariate definitions were dependent on treatment group. For example, the non-EBP group is censored when they initiate CPT/PE; therefore, post-baseline CPT/PE cannot be a covariate in the model. Similarly, for those receiving CPT (PE), post-baseline PE (CPT) and non-EBP individual psychotherapy visits were not included as predictors of censoring.

**S5.** **Sensitivity Analysis using Cloning/Censoring/Weighting Analytic Approach**

Figure S5A. Sample selection for sensitivity analyses using the analytical approach of cloning/censoring/weighting

Table S5A. Baseline Characteristics of All Eligible Person-Trials (Pooled).

| **Characteristic** | **All Eligible Person-Trials (N=89,532)** |
| --- | --- |
| **Sociodemographic data** |  |
| Age, mean (SD), y | 37.44 (9.43) |
| Female, No. (%) | 9014 (10.1%) |
| Racial Category, No. (%) |  |
| White | 65868 (73.6%) |
| Black | 13388 (15.0%) |
| American Indian or Alaska Native | 1097 (1.2%) |
| Asian | 1743 (1.9%) |
| Native Hawaiian or other Pacific Islander | 1646 (1.8%) |
| Multi-racial | 1195 (1.3%) |
| Unknown | 4595 (5.1%) |
| Ethnicity, No. (%) |  |
| Hispanic or Latino | 11817 (13.2%) |
| Not Hispanic or Latino | 73988 (82.6%) |
| Other | 2627 (2.9%) |
| Unknown | 1100 (1.2%) |
| PCL at Baseline (z-score), mean (SD) | -0.00 (1.00) |
| Comorbid Conditions, No. (%) |  |
| Alcohol Abuse | 11897 (13.3) |
| Alcohol Dependence | 10705 (12.0) |
| Depression | 48422 (54.1) |
| Drug Abuse | 6702 (7.5) |
| Drug Dependence | 6565 (7.3) |
| Psychosis | 27168 (30.3) |
| Pain | 46329 (51.7) |
| Suicidal Ideation/Attempt | 6974 (7.8) |
| Traumatic Brain Injury | 12673 (14.2) |

Table S5B: Treatment Effect of Evidence-Based Psychotherapy for PTSD in the Veterans Health Administration, Estimated Using the Cloning/Censoring/Weighting Approach

| **Emulated Trial** | **Initiation/ITT Effect* (95% CI)**** | **Initiation Effect on PCL-M scale (for reference)** | **Completer Effect* (95% CI)**** | **Completer Effect on PCL-M scale (for reference)** |
| --- | --- | --- | --- | --- |
| CPT vs. non-EBP | -0.130 (-0.16, -0.10) | -1.72 (-2.12, -1.32) | -0.377 (-0.386, -0.297) | -4.98 (-5.10, -3.93) |
| PE vs. non-EBP | -0.323 (-0.372, -0.274) | -4.27 (-4.92, -3.63) | -0.538 (-0.585, -0.410) | -7.12 (-7.74, -5.42) |
| PE vs. CPT individual | -0.183 (-0.236, -0.140) | -2.42 (-3.12, -1.86) | -0.249 (-0.801, -0.194) | -3.29 (-10.59, -2.57) |

*Effect is difference between treatment and control group in change in PCL scores, adjusted for baseline PCL z-score, and weighted by inverse probability of censoring and inverse probability of follow-up. A negative value means treatment group had greater improvement (decrease) in symptoms than the control group, e.g., CPT completers had a 0.377 greater decrease in z-score compared to the non-EBP group.

**95% CI estimated with non-parametric bootstrap based on 500 samples.

**Supplement References**

Cornia, R., Patterson, O. V., Ginter, T., & DuVall, S. L. (2014). Rapid NLP Development with LEO. AMIA Annual Symposium. Washington D.C.

Ferrucci, D. & Lally, A. (2004). UIMA: An architectural approach to unstructured information processing in the corporate research environment. *Natural Language Engineering*, *10*(3-4), 327–348.

Willems, S. J. W., Schat, A., van Noorden, M. S., & Fiocco, M. (2018). Correcting for dependent censoring in routine outcome monitoring data by applying the inverse probability censoring weighted estimator. *Statistical Methods in Medical Research*, *27*(2), 323–335. doi:10.1177/0962280216628900
